# Effects of violet light exposure on aging dogs: a pilot study

**DOI:** 10.3389/fvets.2026.1890157

**Published:** 2026-10-16

**Authors:** Kana Miyamoto, Nanami Ono, Shinichiro Kondo, Yukihiro Miwa, Kazuo Tsubota, Makiko Ozawa, Takefumi Kikusui

**Affiliations:** 1 Laboratory of Human-Animal Interaction and Reciprocity, School of Veterinary Medicine, Azabu University, Sagamihara, Kanagawa, Japan; 2 Gunma Animal College, Maebashi, Gunma, Japan; 3 Tsubota Laboratory, Inc., Tokyo, Japan; 4 Aichi Animal Eye Clinic, Nagoya, Aichi, Japan; 5 Department of Ophthalmology, Keio University School of Medicine, Tokyo, Japan; 6 Department of Veterinary Nursing, College of Bioresource Sciences, Nihon University, Fujisawa, Kanagawa, Japan

**Keywords:** aged dogs, cognitive dysfunction syndrome, light therapy, non-invasive intervention, violet right

## Abstract

Aging in dogs is known to induce various physical and brain alterations similar to dementia in humans. Concurrently with the recent extension of canine life expectancy, these age-related changes have imposed growing burdens on both dogs and their owners, emerging as a significant social issue. However, no curative treatment has yet been established, and the current mainstay of management focuses on improving the quality of life through symptomatic therapies, such as environmental modifications and pharmacotherapy. This study focused on violet light, which has suggested efficacy against such age-related changes. We developed a novel violet light irradiation device and evaluated its efficacy and safety in elderly dogs as a pilot study. An intra-individual comparison using an ABA design was employed with geriatric dogs aged 14 to 16 years housed in a senior dog care facility (two of which discontinued midway). The study design consisted of a one-month violet light exposure phase preceded and followed by two-week non-exposure periods. Physical activity levels were measured using accelerometers and cognitive function was assessed using the DISHAA and CCDR. To evaluate the safety, general physical and ophthalmic examinations were conducted by veterinarians. As a result, no significant overall effect was found on activity levels (Daytime: *x*^2^ = 4.22, *p* = 0.12; Nighttime: *x*^2^ = 1.58, *p* = 0.45). Similarly, no significant overall effects were observed for either the DISHAA or the CCDR (DISHAA: *x*^2^ = 1.60, *p* = 0.45; CCDR: *x*^2^ = 1.56, *p* = 0.46). However, a trend toward cognitive improvement was observed in six out of seven dogs and an upward trend in activity levels-indicating physical function improvement-was noted in two out of seven dogs. Regarding safety, no overt clinical abnormalities associated with violet light irradiation were identified. This pilot study demonstrated no clear overall effect, though some individuals showed signs of improvement. Future research should consider large-scale trials controlling for various variables.

## 1 Introduction

Aging in dogs is known to induce various physiological and cerebral changes (1). The brain, in particular, exhibits a wide range of pathological alterations, including significant cerebral atrophy, diffuse plaque deposition of amyloid in the cerebral cortex, cerebral amyloid angiopathy (CAA), and the proliferation of astrocytes and microglia (2, 3). Against the backdrop of these diverse age-related changes in the brain, some dogs develop Cognitive Dysfunction Syndrome (CDS), a disorder characterized primarily by behavioral impairments (4). Typical clinical manifestations of CDS include disorientation, alterations in social interaction and sleep–wake cycle, wandering and anxiety behaviors (5). Canine Cognitive Dysfunction Syndrome (CDS) is defined as a state where these cerebral and behavioral changes have progressed to a severe stage (5). Diagnosis is primarily based on exclusion; CDS is diagnosed when the behavioral changes are prominent after ruling out neurological or endocrine disorders, as well as other conditions that present with similar behavioral alterations. Although behavioral tests such as the Delayed Non-Matching-to-Position (DNMP) task (6) have been developed to assess cognitive function as diagnostic tools, they are infrequently implemented in busy clinical settings. Consequently, diagnosis is often reached through owner interviews (7). To facilitate efficient early diagnosis in clinical practice, various CDS scales have been developed (5, 8, 9). Furthermore, although Magnetic Resonance Imaging (MRI) is the only diagnostic imaging modality available for CDS (10, 11), its clinical application is limited by concerns regarding general anesthesia, high costs, and its relatively low potential to contribute significantly to treatment planning. Consequently, diagnosis currently relies primarily on the evaluation of behavioral changes.

Due to the recent increase in canine life expectancy, CDS has become a significant burden for both dogs and their owners, emerging as a growing societal issue (12). Questionnaire-based surveys designed to assess the severity of CDS have reported that 3.4% of dogs aged 10–12 years, 18.6% of those aged 12–14 years, and 31% of those over 14 years meet the criteria for CDS (8). Although early detection and intervention are desirable for clinical improvement, there are currently few validated biological diagnostic markers in practical use and a curative treatment for the condition has yet to be established. Consequently, the current mainstream approach focuses on slowing disease progression and improving quality of life through pharmacological interventions such as selegiline and propentifylline (5), as well as dietary therapy, nutritional supplementation (13), and environmental enrichment (14).

Violet light is a component of solar radiation, characterized as visible purple light within the wavelength range of 360–400 nm. In this study, this is defined as violet light, which is hereafter abbreviated as VL. Clinical evidence suggests that increased time spent outdoors correlates with a suppressed prevalence and progression of myopia (15, 16), with VL exposure specifically identified as a key contributing factor (17).

It has been demonstrated that the effects of VL exposure are mediated by OPN5, a non-visual, VL-sensitive photoreceptor (18–20). VL can influence the central nervous system through ocular photoreceptive pathways. Mouse studies have also implicated an OPN5-dependent violet-light-sensitive hypothalamic POA pathway (21), although whether a comparable direct POA pathway is engaged in canines remains to be determined. VL has been shown to influence circadian rhythm regulation, mood stabilization, thermogenic regulation, and skin-related photobiological effects (21–24). Furthermore, studies using aged mice have revealed that VL exposure improves memory and increases hippocampal neuronal activity, leading to improvements in cognitive function and depressive-like behaviors (25).

OPN5 is recognized as a receptor that is widely conserved across mammals (26), and the OPN5 gene has also been identified in the canine genome (Ensembl ID: ENSCAFG00845006263). Although VL is present in natural sunlight, it is blocked by conventional indoor lighting and window glass, and a deficiency of VL in indoor environments has been reported (27). Therefore, we hypothesized that VL supplementation could exert beneficial effects via OPN5 activation, even in dogs kept indoors.

In this study, we developed a VL irradiation device as a novel, non-invasive method to alleviate or improve brain aging and CDS in dogs, and subsequently evaluated its efficacy and safety as a pilot study. We hypothesized that VL exposure could mitigate the age-related changes observed in dogs. Specifically, based on reports that dogs with more severe CDS tend to have shorter nighttime sleep and longer daytime sleep (28, 29), we hypothesized that exposure to VL could reduce nighttime awakenings (waking up mid-sleep) and increase daytime activity, thereby leading to clinical improvement in CDS.

## 2 Materials and methods

### 2.1 Subjects

The subjects were 7 aged dogs (4 males, mean age 15.5 ± 0.71 years) housed at Animal Care House (JAPELL Co., Ltd., Aichi, Japan), a specialized nursing facility for elderly dogs (Table 1). Staff selected active individuals with no visual impairments from the facility’s residents, and informed consent was obtained from the owners. ID 1 completed a total of three experimental sessions. ID 4 and ID 6 were excluded from the statistical analysis due to discontinuation caused by poor physical condition. The dogs were fed twice daily (morning and evening) and provided with two exercise sessions per day. All experimental procedures were approved by the Animal Ethics Committee of Azabu University (#230317–4).

**Table 1:** List of the subjects.

ID	Sex	Age	Breed
1	Female	15^a^	Toy Poodle
2	Male	16	Shiba
3	Male	16	Shiba
4^b^	Female	16	Toy Poodle
5	Male	14	Shiba
6^b^	Male	15	Shiba
7	Female	16	Mix

^a^Age 16 at the third session, ^b^Subject excluded statical analysis.

### 2.2 Methods

To account for the high variability among senior dogs and to use each subject as its own control, the study utilized an ABA design over a total duration of approximately 2 months. This included a one-month violet light exposure phase (Light), preceded and followed by 2-week periods without exposure (Pre and Post, respectively) (Figure 1). Mealtimes, exercise routines, and medication schedules for pre-existing conditions were maintained throughout the study. Before enrollment, all dogs underwent body weight measurement, a general physical examination, and a general ophthalmic examination. Dogs with no apparent decrease in appetite or activity were eligible for inclusion. Dogs with systemic or ophthalmic abnormalities considered incompatible with participation in the study were excluded. Dogs with mild pre-existing conditions considered unlikely to affect the study, such as mild mitral regurgitation or an eyelid mass, were eligible for inclusion. An individual dog was withdrawn from the study if the facility staff or veterinarian observed a significant decline in health. Health status was evaluated based on periodic body weight measurements and daily observations of appetite, activity level, and general condition by the facility staff. A veterinary examination was performed if any deterioration in health was suspected. All procedures were conducted at Animal Care House (JAPELL Co., Ltd., Aichi, Japan). Except for general physical and ophthalmic examinations, all interventions were carried out by the facility staff. Recruitment of the subjects began in June 2023, and a total of six cycles were performed between July 2023 and March 2025. While the first and second cycles (ID 1–1, 2–1, 2–2) utilized one-month pre- and post-observation periods, many aged dogs experienced health declines during the three-month schedule. Consequently, from the third cycle onward, the pre- and post-periods were shortened to 2 weeks, and the analysis period was adjusted accordingly. During the study, activity levels were measured using accelerometers, and questionnaires were used to assess cognitive function. Additionally, physical and ophthalmic examinations were performed to ensure the safety of violet light exposure.

**Figure 1:**
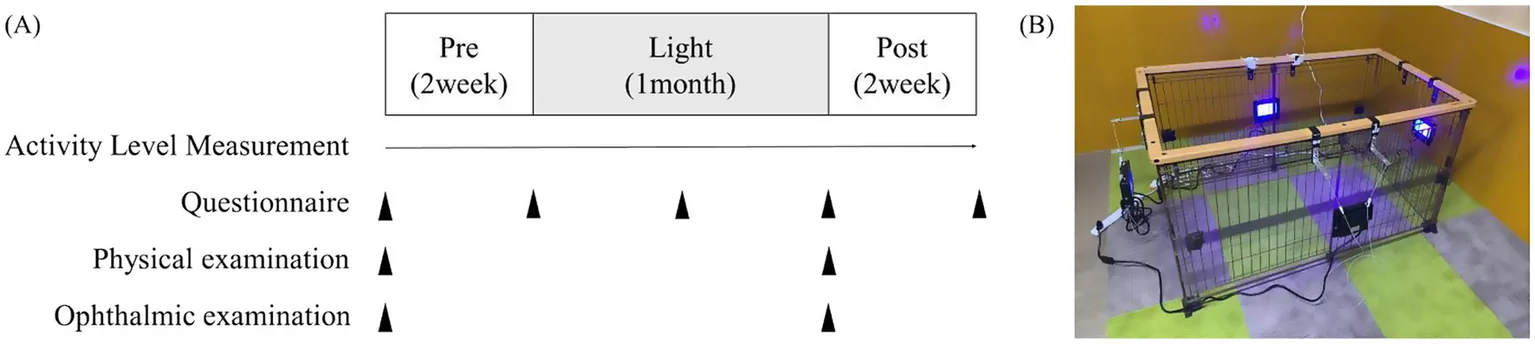
Experimental overview. **(A)** Experimental schedule and assessment items. Experimental schedule consists of 1 month of violet light exposure (Light) and 2 weeks without violet light exposure before and after (Pre and Post). **(B)** Violet light exposure environment.

The violet light exposure period lasted for 1 month (Figure 1), during which the dogs were exposed for 2 hours daily (11:00–14:00). Four light sources were mounted on a cage measuring 120 cm (L) × 73 cm (W) × 70 cm (H) (W-wan circle, Bonbi Alcon Co., Ltd., Osaka, Japan), and the dogs remained inside the cage during the designated hours (Figure 1B). For the first and second cycles (ID 1–1, 2–1, 2–2), a device with a peak wavelength of 400 nm (SUNPIE) was used. From the third cycle onward, a device with a peak wavelength of 375 nm (Contec Co., Ltd., Osaka, Japan) was employed.

Activity levels were measured using a 3-axis Bluetooth accelerometer (MetamotinRL, Mbientlab Co., Ltd., CA, United States) which were attached to the dogs’ collars and Android smartphones (Motorola, SHARP, Co., Ltd., Tokyo, Japan). Measurements were taken continuously 24 h a day during the experimental period, except during charging (Figure 1A). The accelerometer’s sampling rate was set to 12.5 Hz, and data was transmitted to the smartphones via Bluetooth. Following the procedures of a previous study (30), the raw acceleration data were processed using MATLAB (MathWorks, Co., Ltd., Natick, MA, United States; RRID: SCR_001622). The three-axis acceleration data were first combined into a scalar value, and the standard deviation (SD) was calculated every 30 s. Based on these SD values, an automated classification system using a MATLAB-based decision tree categorized the activity levels into seven clusters for each 30-s interval, where higher scores (cluster numbers) indicate greater activity levels.

Cognitive function assessments were conducted on the first day of the pre-period, the first day of the exposure (light) period, 2 weeks after the start of exposure, the first day of the post-period, and the last day of the post-period to investigate temporal changes in detail (Figure 1A). The Canine Cognitive Dysfunction Rating Scale (CCDR) (8) and the DISHAA scale (5) were used for the evaluation, and facility staff completed the questionnaires.

Physical and ophthalmic examinations were performed by a veterinarian twice: at the beginning of the pre-period and at the beginning of the post-period (Figure 1A). The physical examination included measurements of body temperature, heart rate, respiratory rate, and body weight, as well as cardiac auscultation, assessment of oral mucous membrane color, palpation of the peripheral lymph nodes, and abdominal palpation. The ophthalmic examination included assessment of the palpebral reflex, menace response, dazzle reflex, direct and consensual pupillary light reflexes, intraocular pressure measurement, the Schirmer tear test I (STT-1) performed without topical anesthesia, fluorescein staining, and fundus examination.

We used the CONSORT reporting guideline (31) to draft this manuscript, and the CONSORT reporting checklist (32) when editing.

### 2.3 Statistical analysis

All statistical analyses were performed using JMP ver. 14.2.0 (SAS Institute Inc., NC, United States; RRID: SCR_014242). For activity level analysis, data from the 14-day pre-period, the 28 days following the start of exposure (light period), and the 14-day post-period were used. For the first and second cycles, data from the same corresponding periods were extracted and analyzed.

For intra-individual comparison, processed activity data (categorized into seven levels every 30 s) were used to calculate the mean value for each 30-s interval over 7-day periods. These were further averaged for the pre, light, and post periods. The data were then segmented into specific time blocks: day (8:00–20:00), night (20:00–8:00), morning (8:00–12:00), and afternoon (14:00–18:00), and compared using the Friedman test. For variables showing significant differences, the Wilcoxon signed-rank test was performed as a post-hoc analysis, with the Holm method applied for multiple comparison correction.

Next, an overall comparison was conducted using the median values of the mean activity data calculated for the pre, light, and post periods in the intra-individual analysis. The median values for each period from the seven subjects were categorized—consistent with the intra-individual comparison - into day, night, morning, and afternoon segments, and compared using the Friedman test. For variables showing significant differences, the Wilcoxon signed-rank test was performed as a post-hoc analysis, with the Holm method applied for multiple comparison correction.

For questionnaire analysis, the first and second assessments were designated as the pre-period, the third and fourth as the light-period, and the fifth as the post-period. For the first and second cycles, data from the same corresponding periods were extracted and analyzed. The CCDR score was determined by first calculating the mean of the response scores for each question. Because some situations queried in the questionnaire did not occur during the experimental period, resulting in missing responses, the mean was utilized instead of the total score. Furthermore, because there were no missing data in either questionnaire for the first baseline response, the severity of CDS was determined by the sum of the scores, based on previous studies (5, 8). Thereafter, the average scores for each period were calculated, and comparisons among the periods were performed using the Friedman test. Where significant differences were found, the Wilcoxon signed-rank test was performed as a post-hoc analysis, with the Holm method applied for multiple comparison correction. Similar to the CCDR, the mean of the response scores for each question was used for the analysis of the DISHAA. Both the mean total scores and the mean scores for individual items were calculated for each period. Similar to the CCDR analysis, these were compared using the Friedman test, followed by the Wilcoxon signed-rank test as a post-hoc analysis with Holm correction.

Because ID 1 was measured repeatedly, a generalized linear mixed model (GLMM) was also applied to the pooled data from all dogs to account for this effect. Due to the small sample size, no covariates were included; the experimental period (pre, light, and post) was treated as the explanatory variable, and the individual ID was included as a random effect.

Additionally, statistical analyses for both activity levels and questionnaires were conducted excluding two subjects with severe cognitive dysfunction (ID 2–1 and 2–2). The exclusion criteria were defined as a total score of 50 or higher on the CCDR and 34 or higher on the DISHAA at the first assessment.

## 3 Results

### 3.1 Overall results

Comparisons of activity levels across different time periods (day, night, morning, afternoon) and cognitive questionnaire scores using the Friedman test revealed no significant differences (Table 2; Figure 2). Similarly, no significant variations were found in individual DISHAA items. Even after excluding the two dogs with severe cognitive dysfunction (ID 2, 3) and re-analyzing the data, no differences were observed. Additionally, to account for the multiple experimental sessions conducted for ID 1, a generalized linear mixed model (GLMM) was performed with ID included as a random effect; however, no significant differences were observed for any of the items (Supplementary Table S1). Furthermore, physical and ophthalmic examinations showed no clinical abnormalities that would significantly affect cognitive function or activity levels.

**Table 2:** Statistical comparison of activity level and questionnaire scores among periods using the Friedman test (*n* = 7).

Variable		x^2^ (df = 2)	*p*-value
Activity level	Daytime	4.22	0.12
	Nighttime	1.58	0.45
	Morning	2.29	0.31
	Afternoon	2	0.36
Questionnaire	CCDR	1.56	0.46
	DISHAA	1.60	0.45

**Figure 2:**
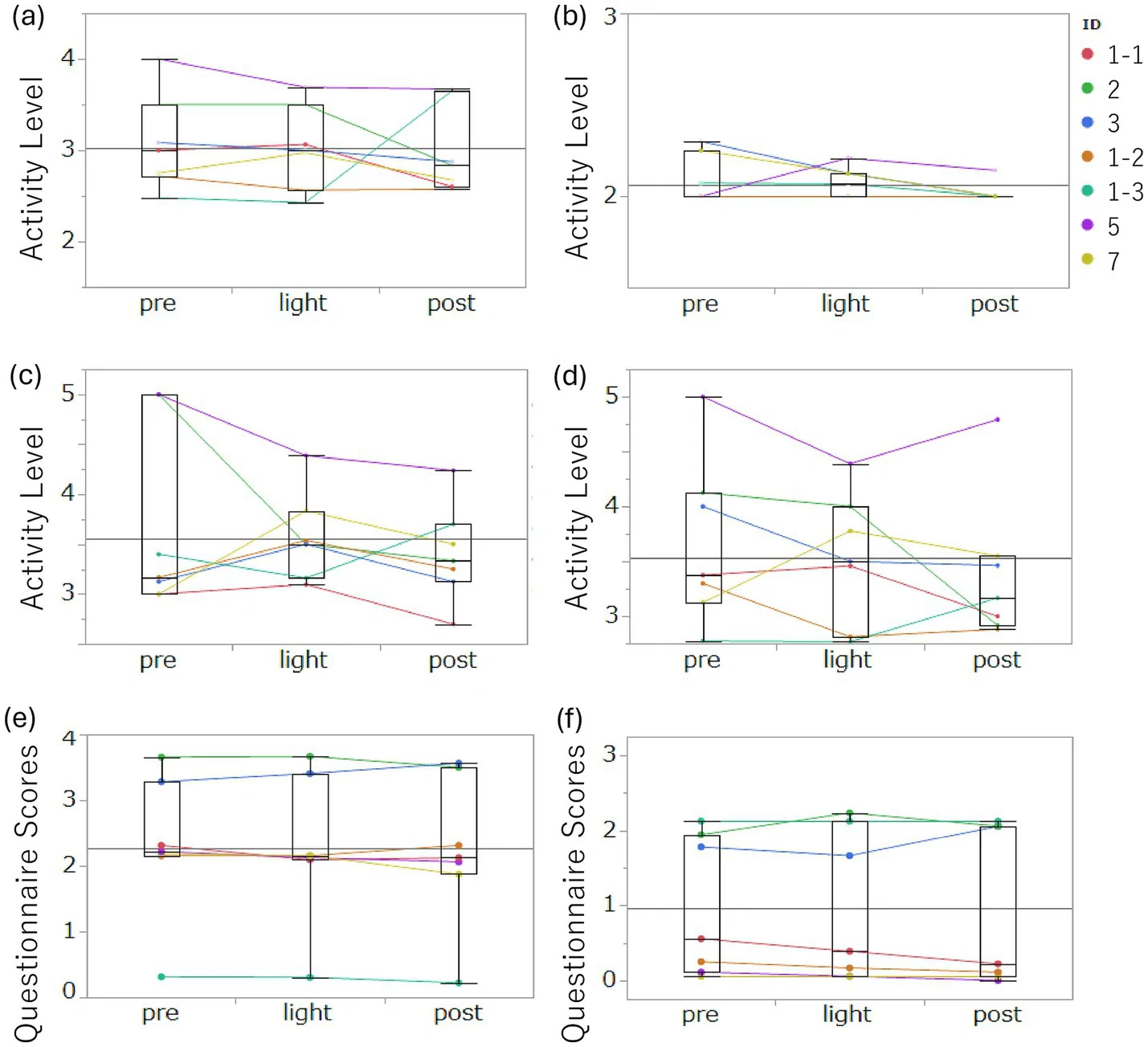
Comparison of activity level and questionnaire scores among periods. **(a)** Activity level of daytime, **(b)** Activity level of nighttime, **(c)** Activity level of morning, **(d)** Activity level of afternoon, **(e)** Questionnaire scores of Canine Cognitive Dysfunction Rating Scale, **(f)** Questionnaire scores of DISHAA.

### 3.2 Individual results

The activity levels and cognitive questionnaire scores are presented in Table 3 and Figure 3, respectively. Since ID 1 completed a total of three experimental sessions in order to examine the consistency of the effects of VL in the same individual, and individual variation in the effects of VL according to the stage of ageing, the data from the first, second, and third sessions are designated as ID 1–1, ID 1–2, and ID 1–3, respectively.

**Table 3:** Statistical comparison of activity levels among three experimental periods (Pre, Light, Post) for each dog using the Friedman test and Wilcoxon signed-rank test.

ID	Time of day	Median			X^2^ (df = 2)	*p*-value	Pre-Light	Pre-Post	Light-Post
		Pre	Light	Post					
1–1	Daytime	3.5	3	2.7	76.83	<0.0001	0.0015	0.0003	0.0003
	Nighttime	3	3	2.7	0.43	0.8			
	Morning	3	3.1	3.6	2.28	0.31			
	Afternoon	3.4	3.5	3	107.44	<0.0001	0.0528	0.0002	0.0003
2	Daytime	3.5	3.5	2.8	3.56	0.16			
	Nighttime	2	2	2	0.0002	0.99			
	Morning	5	3.5	3.3	25.62	<0.0001	0.0003	0.0002	0.0528
	Afternoon	4.1	3.5	2.9	157.15	<0.0001	0.0003	0.0002	0.0001
3	Daytime	3.1	3	2.9	80.97	<0.0001	0.7412	0.0003	0.0002
	Nighttime	2.3	2.1	2	148.2	<0.0001	0.0014	0.0003	0.0002
	Morning	3.1	3.5	3.1	159.08	<0.0001	0.6789	0.0002	0.0002
	Afternoon	4	3.5	3.5	74.76	<0.0001	0.0003	0.0002	0.9586
1–2	Daytime	2.7	2.6	2.6	36.82	<0.0001	0.0003	0.0002	0.4852
	Nighttime	2	2	2	0.72	0.69			
	Morning	3.2	3.5	3.3	12.91	0.0016	0.0003	0.0467	0.0820
	Afternoon	3.3	2.8	2.9	128.45	<0.0001	0.0003	0.0002	0.0001
1–3	Daytime	2.8	2.7	2.9	66.35	<0.0001	0.0003	0.0002	0.0001
	Nighttime	2.1	2.1	2.1	2.46	0.29			
	Morning	3.4	3	3.4	220.57	<0.0001	0.0003	0.0002	0.0001
	Afternoon	2.9	2.9	3.2	95.37	<0.0001	0.0295	0.0003	0.0002
5	Daytime	3.9	3.7	3.7	128.74	<0.0001	0.0003	0.0002	0.0780
	Nighttime	2.1	2.3	2.2	252.97	<0.0001	0.0003	0.0002	0.0001
	Morning	4.3	4	4.1	183.55	<0.0001	0.0003	0.0002	0.0001
	Afternoon	4.7	4.4	4.5	114.7	<0.0001	0.0003	0.0002	0.0001
7	Daytime	3	3.2	2.9	252.19	<0.0001	0.0003	0.0002	0.0001
	Nighttime	2.2	2.2	2.1	58.07	<0.0001	0.0003	0.0002	0.0001
	Morning	3.5	3.6	3.3	64.31	<0.0001	0.0060	0.0020	0.0001
	Afternoon	3.1	3.8	3.4	100.9	<0.0001	0.0003	0.0002	0.0001

*Post-hoc* *p*-values were adjusted using the Holm method.

**Figure 3:**
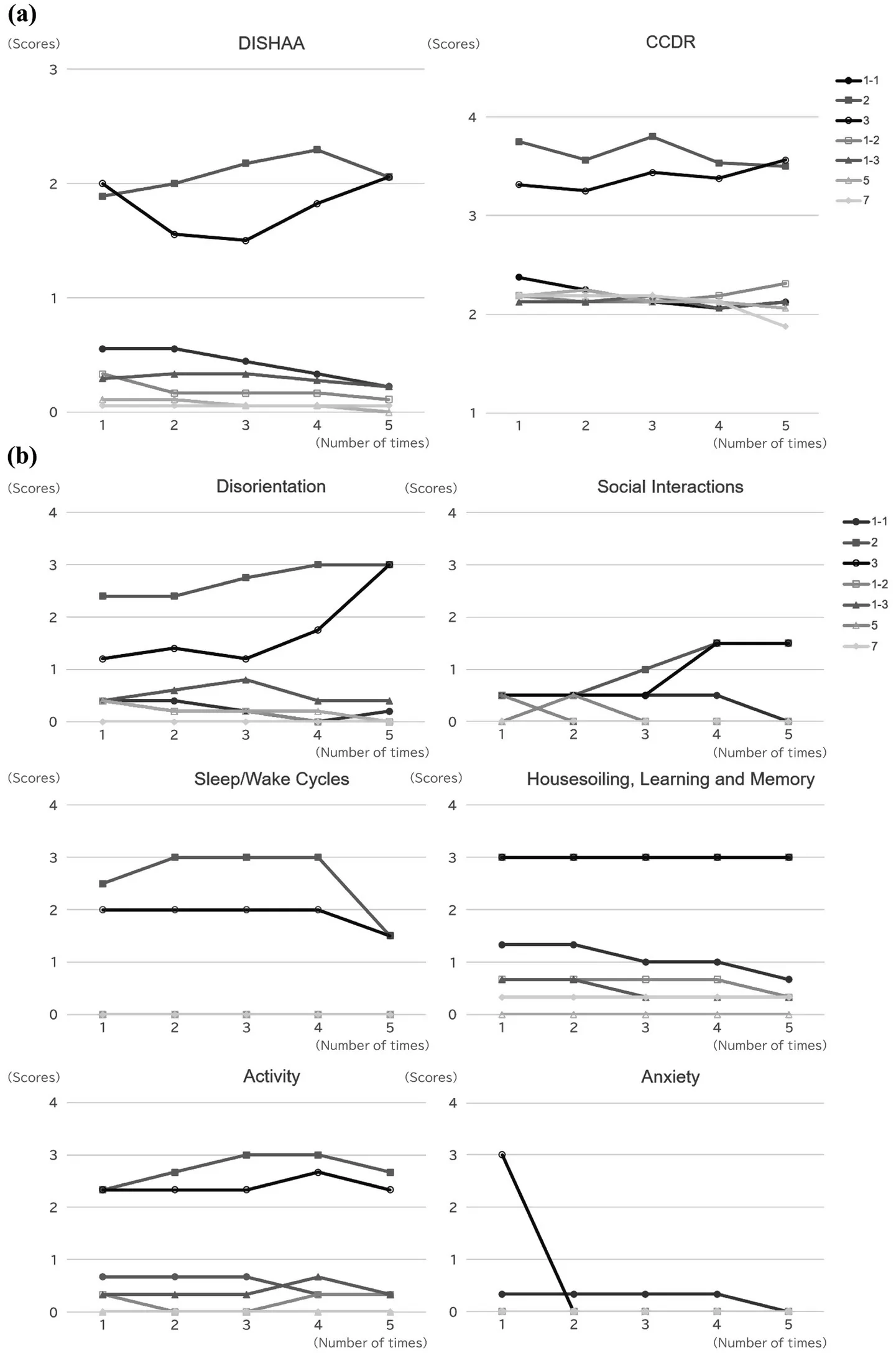
Changes in questionnaire scores for each individual. **(a)** Total scores. **(b)** DISHAA scores by category.

Regarding the activity levels of ID 1–1, significant differences were observed between pre-light, pre-post, and light-post during the daytime, as well as between pre-post and light-post in the afternoon. Daytime activity levels showed a progressive decrease over time, while afternoon activity was higher in the pre and light periods compared to the post period (Table 3). The total DISHAA score decreased from pre to light and post periods; at the item level, similar trends were observed in house soiling, learning and memory, and activity (Figure 3). The total CCDR score was highest at pre and decreased in the light and post periods compared to the pre period (Figure 3).

In the case of ID 2, significant differences in activity levels were observed between pre-light and pre-post during the morning, and between pre-light, pre-post, and light-post in the afternoon. Morning activity was higher in the pre period compared to the light and post periods, while afternoon activity showed a progressive decrease over time (Table 3). The total DISHAA score increased toward the latter half of the light period and decreased in the post period (Figure 3). At the item level, disorientation and social interactions scores increased chronologically, sleep/wake cycles scores decreased in the post period, and activity scores were highest during the light period (Figure 3). The total CCDR score increased during the first half of the light period and decreased from the second half of the light period through the post period (Figure 3).

Analysis of the activity levels for ID 3 revealed, significant differences between pre-post and light-post during the daytime; pre-light, pre-post, and light-post at night; pre-light and light-post in the morning; and pre-light and pre-post in the afternoon (Table 3). Daytime activity was higher in the pre and light periods compared to the post period, while nighttime activity showed a progressive decrease over time (Table 3). Morning activity peaked in the light period compared to the pre and post periods, and afternoon activity was higher in the pre period than in the light and post periods (Table 3). The total DISHAA score was lowest during the light period (Figure 3). Analysis of individual items revealed that disorientation and social interaction scores increased in the post period, whereas sleep/wake cycle scores decreased in the post period (Figure 3). Activity scores increased chronologically, and anxiety scores were highest during the pre-period. The total CCDR score exhibited a continuous increase over time (Figure 3).

When examining ID 1–2 (ID 1–1, 2nd session), significant differences in activity levelswere observed between pre-light and pre-post during the daytime; pre-light in the morning; and pre-light, pre-post, and light-post in the afternoon (Table 3). Daytime activity was higher in the pre period compared to the light and post periods (Table 3). Morning activity peaked in the light period compared to the pre and post periods. Afternoon activity was higher in the pre period than in the light and post periods; notably, it was also higher in the post period compared to the light period (Table 3). The total DISHAA score showed a chronological decrease, with a similar trend observed in the disorientation score at the item level (Figure 3). Although the total CCDR score was highest during the post period, no substantial changes were observed overall (Figure 3).

The activity profile of ID1-3 (ID 1–1, 3nd session) demonstrated, significant differences between pre-light and pre-post during the daytime; across all intervals (pre-light, pre-post, and light-post) in the morning; and between pre-post and light-post in the afternoon (Table 3). Daytime activity was higher in the pre period than in the light period, with the post period being higher than both the pre and light periods (Table 3). Morning and afternoon activity levels exhibited similar trends to those observed during the daytime (Table 3). No significant changes were found in the total DISHAA score (Figure 3). However, item-level analysis revealed that the disorientation score was highest during the light period, while scores for house soiling, learning and memory were highest during the pre-period (Figure 3). Similarly, the total CCDR score showed no substantial variations (Figure 3).

With respect to ID 5, significant differences in activity levelswere observed between pre-light and pre-post during the daytime; across all intervals (pre-light, pre-post, and light-post) at night; between pre-post and light-post in the morning; and across all intervals in the afternoon (Table 3). Daytime activity was higher in the pre period than in the light and post periods, while nighttime activity was highest in the light period, with the post period being higher than the pre period (Table 3). Morning activity levels exhibited similar trends to those observed during the daytime (Table 3). In the afternoon, activity was higher in the pre period than in the light and post periods, with the post period being higher than the light period (Table 3). The total DISHAA score showed a progressive decrease over time (Figure 3). Item-level analysis revealed that the disorientation score exhibited similar trends to the total score (Figure 3). Similarly, the total CCDR score exhibited a chronological decrease throughout the study (Figure 3).

Finally, focusing on ID 7, significant differences in activity levels were observed across all intervals (pre-light, pre-post, and light-post) during the daytime; across all intervals at night; between pre-post and light-post in the morning; and across all intervals in the afternoon (Table 3). Daytime activity was higher in the pre and light periods compared to the post period (Table 3). Nighttime activity was higher in the light period than in the pre period, and higher in both the pre and light periods than in the post period (Table 3). Morning activity levels exhibited similar trends to those observed during the daytime. In the afternoon, activity was higher in the pre period than in the light and post periods, with the light period being higher than the post period (Table 3). No significant changes were found in either the total DISHAA score or the individual item scores (Figure 3). Similarly, the total CCDR score exhibited a chronological decrease throughout the study (Figure 3).

In Table 4, the areas where a trend toward improvement was observed following VL exposure are indicated by red arrows. Figure 4 shows the diurnal variations for the individuals in whom a potential effect on activity levels was suggested. In ID 3 and 7, the results suggested effects on both activity levels (physical activity) and questionnaire scores (cognitive function). In ID 1–1, 1–2, 1–3, and 5, although no effect on physical function was demonstrated, the questionnaire scores showed a trend toward improvement, suggesting an effect on cognitive function. ID 2 did not show any trend toward improvement.

**Table 4:** Changes in activity levels and questionnaire scores compared with Pre values.

ID	CDS severity	Activity Level								Questionnaire			
		Daytime		Nighttime		Morning		Afternoon		DISHAA		CCDR	
		Light	Post	Light	Post	Light	Post	Light	Post	Light	Post	Light	Post
1–1	Mild	↓	↓	—	—	—	—	—	↓	↓	↓	↓	↓
2	Severe	—	—	—	—	↓	↓	↓	↓	↑	↑	↑	↓
3	Severe	—	↓	↓	↓	↑	—	↓	↓	↓	↑	↑	↑
1–2	Mild	↓	↓	—	—	↑	—	↓	↓	↓	↓	—	↑
1–3	Mild	↓	↑	—	—	↓	↑	↓	↑	—	—	↓	↓
5	Normal	↓	↓	↑	↑	↓	↓	↓	↓	↓	↓	↓	↓
7	Normal	↑	↓	↓	↓	↑	↓	↑	↑	—	—	↓	↓

CDS severity: Baseline severity based on DISHAA classification (5). Activity Level: Individual statistics completed. ↑↓ Significant difference. — Not Significant difference. Questionnaire: Individual Score Changes (Not statistics). Values shown in the shaded cells indicate effects that may be meaningful.

**Figure 4:**
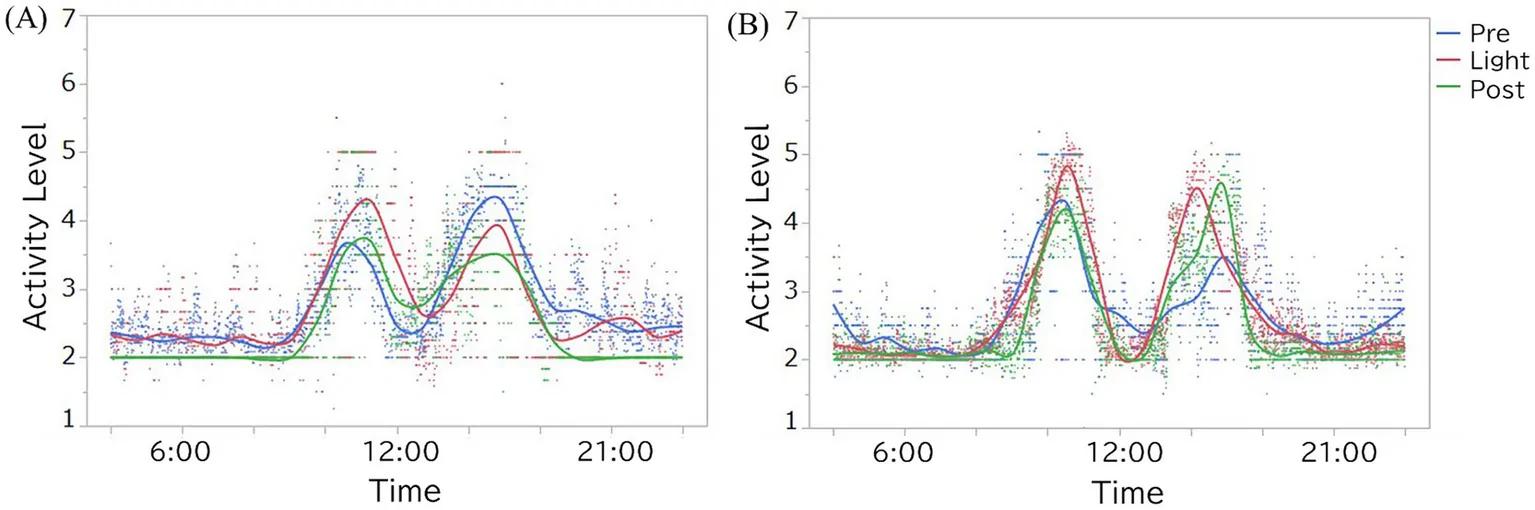
Two cases of diurnal variation suggesting the effect of violet light exposure on activity levels. **(A)** ID 3, Nighttime activity decreased over time. **(B)** ID 7, Daytime activity was high during the light period, while nighttime activity decreased over time.

## 4 Discussion

This study evaluated the therapeutic effects of VL exposure in geriatric dogs. Based on the overall analysis of activity levels and questionnaire scores (Table 2), we were unable to demonstrate a statistically significant global effect of VL exposure. Although no clear effects were observed following exposure to VL, a key strength of this study is that it was conducted under controlled, identical environmental conditions, rather than using privately owned dogs whose household environments are difficult to regulate. Furthermore, to control for background factors such as breed and age, we employed an ABA within-subject design. Additional strengths include evaluations conducted by experienced staff and precise activity measurements utilizing accelerometers. An individual case-by-case analysis revealed a trend toward improvement in several subjects (Table 4). Specifically, six out of seven dogs exhibited a positive trend in questionnaire scores, indicating potential cognitive improvement. In contrast, only two out of seven dogs showed enhanced physical activity levels. These findings suggest that VL exposure may exert distinct effects on physical and cognitive functions independently, implying that changes in physical function and central nervous system function are not necessarily causally linked (33). The mechanisms underlying the observed outcomes remain unclear because OPN5 expression or activation was not directly assessed in the present canine study. OPN5, a non-visual, VL-sensitive photoreceptor, is expressed in the retina, the preoptic area (POA), and the skin (18, 19, 21, 23). Retinal OPN5 has been shown to suppress myopia by inhibiting hyaloid vasculature (17, 20, 34) and to participate in the photoentrainment of circadian rhythms (22). Furthermore, it is known to project to the medial subdivision of the dorsal habenula (25). The medial habenula regulates brain functions such as depression, fear, memory, and anxiety through neural pathways to the interpeduncular nucleus (35), suggesting that VL exposure may modulate these processes. Meanwhile, OPN5 expressed in the POA responds to VL and transmits signals to brown adipocytes via POA neurons (21). Given that the POA primarily governs sleep homeostasis (36, 37) and anxiety regulation (38), VL exposure is expected to exert beneficial effects on these functions as well. However, this interpretation remains speculative, and future studies should directly evaluate OPN5 expression and pathway activation in canines.

Based on these findings, while VL exposure is thought to contribute to improvements in cognitive function, circadian rhythms, and mood, it is unlikely to directly enhance physical function. It is possible that changes in activity levels resulting from an improved sleep–wake cycle were not sufficiently detected by the accelerometers. The analysis method used in this study was insufficient for detecting sleep–wake cycles, suggesting that a more detailed analytical approach needs to be considered in future research.

These findings suggest that indoor-housed dogs may also be susceptible to “indoor diseases.” In humans, a close correlation between outdoor activity and health outcomes—such as the prevalence of myopia and depression—is well-established (39, 40). Our results indicate that, similarly, the lack of outdoor exposure may significantly impact the overall health and quality of life of indoor dogs.

Although VL exposure demonstrated a potential to improve cognitive function in some geriatric dogs, the present study is subject to several limitations.

Some dogs included in this study presented with nuclear sclerosis. Given that OPN5 located in the retinal intrinsically photosensitive retinal ganglion cells (ipRGCs) is considered one of the primary sites of action for VL, individual variations in ocular light transmittance may have influenced the treatment efficacy (41). While nuclear sclerosis is a naturally occurring age-related change in dogs - making it challenging to recruit subjects entirely free of the condition - future studies could mitigate these discrepancies by selecting subjects with as uniform an ocular status as possible to standardize light transmittance.

In this study, the VL exposure was administered between 11:00 and 14:00. This timeframe corresponds to a period of lower activity within the facility’s routine; notably, since the subjects were geriatric dogs, they tended to spend extended periods resting with their eyes closed, even during daylight hours, due to age-related declines in activity. Consequently, it is possible that some subjects were asleep during the exposure sessions. Given that VL efficacy requires the subjects to have their eyes open for the light to reach the retina, future protocols should prioritize exposure during periods of confirmed wakefulness, such as during feeding. Furthermore, although the devices were positioned on all four sides of the cages, the brightness may have caused the dogs to squint or close their eyes. Optimizing both the timing of irradiation and the method of light delivery remains a critical task for future research.

Furthermore, this study evaluated the effects of VL exposure over a relatively short period of approximately 1 month. In clinical trials for human dementia and CDS, interventions are typically administered over 6 months or even years to assess their efficacy in slowing cognitive decline. While the primary objective of this research was to alleviate or improve existing cognitive impairment through VL exposure, future studies should also investigate its potential long-term effects in suppressing the progression of cognitive decline.

Among the subjects in this study, only two met the diagnostic criteria for CDS at baseline, as determined by questionnaire scores. Therefore, these results are insufficient to definitively conclude the efficacy of VL exposure specifically for canine CDS. Future research should focus on a cohort exclusively meeting CDS criteria to evaluate its therapeutic potential. Furthermore, the small sample size precluded comparative analyses based on the severity of cognitive dysfunction, age, or breed. In particular, the cases in this study were heavily biased toward very geriatric dogs. It has been reported that the prevalence of CDS increases with age (8, 42); therefore, the effects of VL irradiation should be investigated in senior dogs, particularly during the pre-onset or early stages of CDS. Furthermore, the baseline characteristics of the subjects in this study, such as breed and body size, were not fully standardized. Given that small breed dogs carry a higher risk of developing CDS (43), and because variations in CDS prevalence associated with breed and neuter status (44, 45) have been previously documented, it is necessary to examine how these differences influence the efficacy of VL irradiation.

In conclusion, while this pilot study did not demonstrate a clear overall effect, the potential efficacy of VL irradiation was observed in a subset of individuals. Furthermore, the study identified improvable limitations within the experimental protocol, including subject selection criteria and VL irradiation methodologies. In the future, optimizing these experimental conditions and conducting larger-scale investigations will allow for a more definitive evaluation of the efficacy of VL irradiation in aged dogs.

## Data Availability

The raw data supporting the conclusions of this article will be made available by the authors upon requests.
