# Supplementary material for: Effects of violet light exposure on aging dogs: a pilot study
Source: Front Vet Sci. 2026 Oct 16;13:1890157. doi: 10.3389/fvets.2026.1890157 (PMC13720361; doi:10.3389/fvets.2026.1890157)
Supplement: Supplementary file 1 [file Table_1.pdf]

Table S1. GLMM results for activity levels and questionnaires with individual ID as a random effect

Variable		β	SE	t	*p* -value
Activity Level (Daytime)	Period [light-pre]	-0.05	0.18	-0.25	0.8
	Period [post-pre]	-0.09	0.18	-0.51	0.62
Activity Level (Nighttime)	Period [light-pre]	-0.01	0.04	-0.33	0.75
	Period [post-pre]	-0.07	0.04	-1.61	0.13
Activity Level (AM)	Period [light-pre]	-0.1	0.24	-0.4	0.7
	Period [post-pre]	-0.26	0.24	-1.08	0.3
Activity Level (PM)	Period [light-pre]	-0.14	0.19	-0.74	0.47
	Period [post-pre]	-0.28	0.19	-1.44	0.17
CCDR	Period [light-pre]	-0.03	0.39	-0.08	0.94
	Period [post-pre]	-0.07	0.39	-0.17	0.87
DISHAA (Total score)	Period [light-pre]	-0.02	0.38	-0.05	0.96
	Period [post-pre]	-0.03	0.38	-0.07	0.94
DISHAA (Disorientation)	Period [light-pre]	0.02	0.21	0.1	0.92
	Period [post-pre]	0.2	0.21	0.95	0.36
DISHAA (Social Interactions)	Period [light-pre]	0.11	0.17	0.63	0.54
	Period[post-pre]	0.14	0.17	0.85	0,41
DISHAA (Sleep/Wake Cycles)	Period [light-pre]	0.04	0.15	0.24	0.82
	Period [post-pre]	-0.25	0.15	-1.65	0.12
DISHAA (Housesoiling, Learning and Memory)	Period [light-pre]	-0.1	0.12	-0.76	0.46
	Period [post-pre]	-0.2	0.12	-1.53	0.15
DISHAA (Activity)	Period [light-pre]	0.1	0.08	1.19	0.26
	Period [post-pre]	0	0.08	0	1
DISHAA (Anxiety)	Period [light-pre]	-0.21	0.17	-1.23	0.24
	Period [post-pre]	-0.26	0.17	-1.5	0.16
